# The evolution of social media in nephrology education: A mini-review

**DOI:** 10.3389/fneph.2023.1123969

**Published:** 2023-02-13

**Authors:** Mythri Shankar, Matthew A. Sparks

**Affiliations:** ^1^ Department of Nephrology, Institute of Nephro-urology, Bengaluru, India; ^2^ Division of Nephrology, Department of Medicine, Duke University School of Medicine, Durham, NC, United States; ^3^ Renal Section, Durham VA Health Care System, Durham, NC, United States

**Keywords:** blogs, Twitter, Facebook, medical education, social media

## Abstract

Social media is defined as “a group of Internet-based applications that build on the ideological and technological foundations of Web 2.0, that allow the creation and exchange of user-generated content”. Social media can be used in medical education to enhance knowledge sharing among peer groups and the public in general. The internet revolutionized learning by allowing easier dissemination of knowledge that did not depend on printing and physical distribution of books, journals, or magazines. According to a report from 2018, 95% of students have access to smartphones and 45% are online at any given time. Social media platforms are powerful tools to spread knowledge by the way of stories, videos, and educational games. Both formal and informal learning can be achieved with the use of social media. The microblogging website Twitter has become a popular social media platform by many in medical education including the nephrology community. Twitter, for example, is used to build communities, discuss journal articles, inform the community of conferences, share infographics and visual abstracts of original research work. As an example, it can be difficult for women in nephrology to connect and travel to make a physical presence. The use of social media allows women to connect *via* webinars and Women in Nephrology (WIN) India live Twitter chats. Thus, social media can help facilitate networking and collaboration with nephrologists all over the world. Social media has limitations as well. Insensitive posts can have a detrimental effect on one’s career. A survey has shown that increased use of social media can contribute to addiction, anxiety, diminished self-esteem, and even depression. Hence, in order to effectively use social media to contribute positively to one’s career, we recommend considering the positive and negative aspects of social media.This review will discuss the various social media platforms and how they have been applied to nephrology education.

## Introduction

The world has continued to embrace digitalization and the internet. Social media platforms such as Facebook, Twitter, Instagram, YouTube, and TikTok are a part of daily routine and thus intertwined in the social fabric of lives. As of 2021, 4.26 billion people were using social media worldwide, which is projected to increase to 6 billion by 2027 (1). Today, 72% of Americans and 47% of Indians use some form of social media (2, 3). Social media has become a de facto town square where unique ideas are discussed, debated, and disparate groups are able to come together and build like-minded communities. Moreover, social media has given a voice, diminished barriers, and resulted in more equitable opportunities. One of the advantages of social media is that it empowers the end user to share, discuss, and educate. Thus, social media has dismantled the traditional hierarchical approach to education, including medical education, where only the select few could teach *via* textbooks, at conferences, or in journal articles. Social media allowed for anyone to join the conversation. An educational needs assessment of US nephrology fellows in 2021 showed diminished use of traditional educational resources and a greater use of digital free open-access medical education (FOAMed) resources from 2016 to 2021 (4). Thus, showing the transformation that has occurred in nephrology education over the last decade.

The ubiquitous spread of smartphones and increasing access to the internet in remote rural areas has allowed medical education *via* social media to spread throughout the world. The coronavirus disease 2019 (COVID-19) pandemic further accelerated the use of social media to share medical information and discuss the fast-paced and ever changing information regarding the pandemic. In parallel, there was an immense increase in the submission of papers to preprint servers which ignited public discussions and peer review on social media platforms like Twitter, blogs, podcasts, and YouTube were the norm (5). However, social media has both positive and negative effects. Here we discuss the influential role of social media in education and its pitfalls.

Social media can empower students, teachers, and patients to build a community and share information. Moreover, social media can allow people to connect and learn from other groups typically siloed in different disciplines and institutions. According to a report, 96% of students with internet access use at least one social media platform for educational purposes (6). The nephrology community has embraced social media over the last several decades (7).

Traditionally, nephrology has been identified as filled with challenging concepts (8). The standard teaching is by didactic lectures, which have formed the backbone of medical education, are often ineffective, passive, and fail to achieve widespread dissemination (9). Active learning techniques have the potential to provide in-depth comprehension and better concept retention (10–12). Bonwell and Eison have defined active learning as “involving students in doing things and thinking about the things they are doing” (12). Social media is one of the promising platforms for implementing active learning in nephrology education.

## Social media

Social media is a collective term for internet-based applications and websites focusing on interaction, communication, sharing content, and collaboration. In the early 2000s, the advent of Web 2.0 allowed users to interact and collaborate in a virtual community as creators of user-generated content. Throughout the years social media platforms have been created to allow individuals to stay in touch with family, friends, and the larger community. Businesses use social media platforms to promote their products and track customer insights. There are various social media platforms with unique attributes. Facebook, launched in 2004, is a social networking website with the largest number of users worldwide. Meta, the umbrella company, now owns Facebook, Instagram, and WhatsApp. Registered users create their profiles, upload photos and videos, and send messages to keep in touch with their community. However, in order to follow an individual on Facebook, both parties must accept the invitation. YouTube, (founded in 2005) a social media platform for individuals to upload original videos. It is currently the second most commonly used platform. WhatsApp (2009) is a multi-platform messaging application that allows users to share photos, videos, text messages, and their status. It works on a wide range of platforms and is an inexpensive way to connect with people across the globe. However, WhatsApp is a closed group of individuals, thus limiting the ability to disseminate information widely. Instagram (2010) is a free photo and video sharing application where registered users share photos or short videos with their followers. They can also view, like, and comment on the photos or short videos shared by their friends. While video, and photos can be shared widely, two-way conversation is limited. TikTok (2011) brands itself as “the leading destination for short-form mobile video” to inspire creativity and spread joy. Pinterest (2010) is a go-to place for rich visuals. Twitter (2006) is a micro-blogging site and undoubtedly the birthplace of nephrology education. Users interact with short messages that have a 280 character limit called “tweets”. The unique aspect to Twitter is that you are able to follow individuals without the other person’s permission (unlike Facebook). Thus, allowing for more diverse followers and instantaneous adding and deleting of one’s social media feed. Twitter allows its users to participate actively or passively in the learning process. Users can passively view others’ tweets or connect and produce their own content. The content is free, real-world, dynamic, original, and diverse. This is directly opposite of a textbook, which is reviewed, oftentimes already outdated, and comes at a cost. The content on Twitter is unpolished making it more real-world, however, users can link to the primary journal articles. Twitter provides an instant audience with multiple users providing feedback on the content posted enriching the learning process (13, 14).

**Figure 1:**
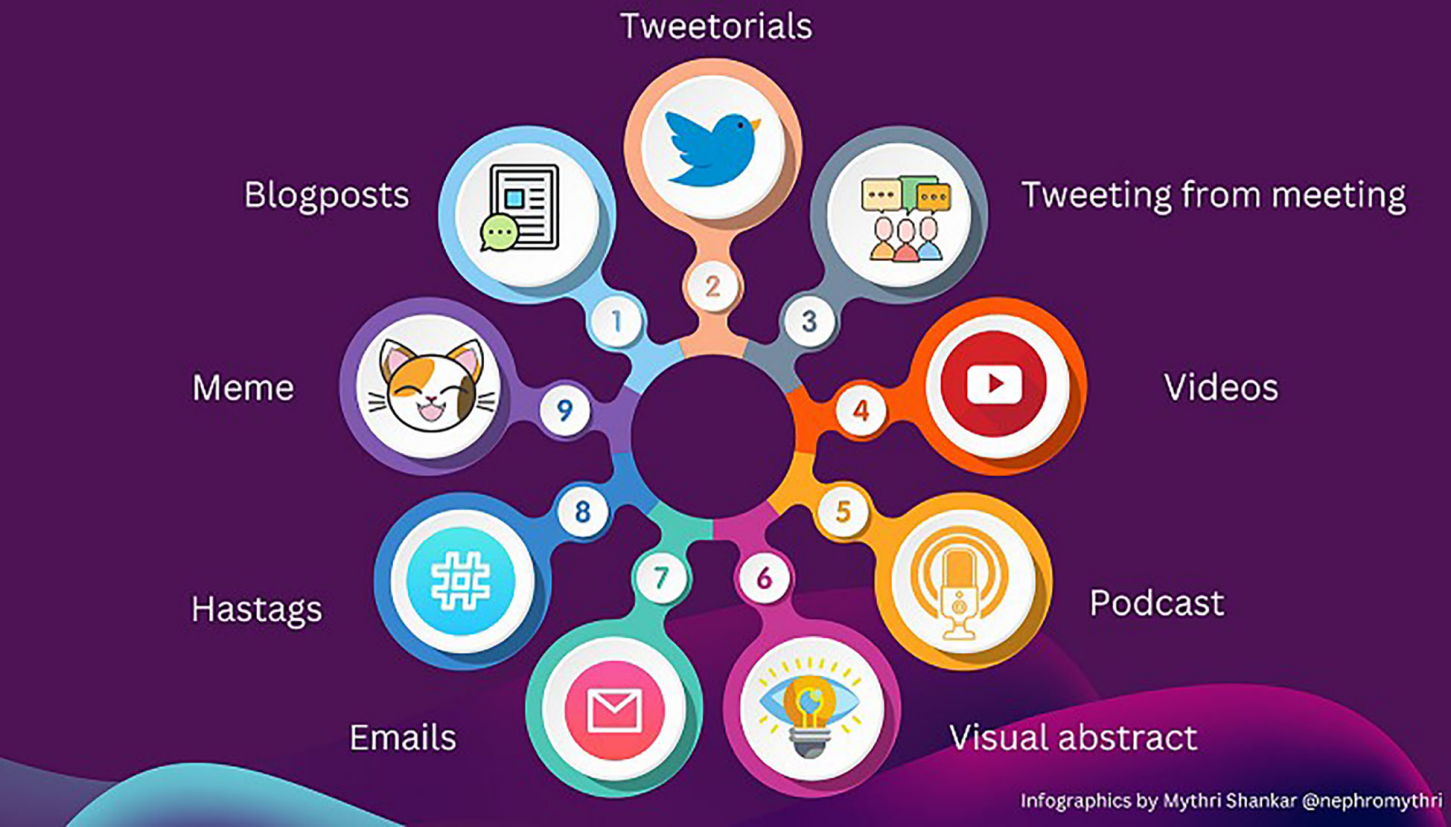
Various modes of social media.

Free open-access medicine education (FOAMed) is the primary product of Web 2.0 with many forms such as discussions (on Twitter), podcasts (audio content), live conference tweeting, visuals (Twitter, Instagram, Facebook, etc), video content (YouTube), and essays (blog posts). Social media also simplifies collaboration among healthcare workers around the world (15) (**Figure 1**).

## Blog posts and Tweetorials

The term “blog” was first coined in 1997 by Peter Merholz who shortened the term web log to blog. A blog is a user generated website used to share short (or long) articles with no publishing paywall or impediment to publishing. Blogs may or may not be peer reviewed. However, the comment section serves as a peer review forum. A blog is a great starting point to keep up with the latest developments and innovations in the field. It allows the user to update the contents by just uploading and attaching files to it. A blog can become interactive if the author allows readers to comment. Posts are archived and organized in reverse chronological order, so the latest ones are visible first to the readers. The Renal Fellow Network was one of the earliest blogs (originally hosted by the Google platform, Blogger now on Wordpress) in nephrology, started by the late Dr. Nathan Hellman in 2008 as a nephrology fellow (16). The content is peer-reviewed by a team of nephrologists and fellows before publication. Glomcon pubs is another blog, where the content focuses primarily on glomerular diseases and is peer-reviewed by editors before publication. Glomcon pubs is an online educational library of blog posts, infographics, and short videos catering to glomerular diseases (17). A few other nephrology blogs of interest for nephrologists are Precious Bodily Fluids by Dr. Joel Topf (18), Nephron Power by Dr. Kenar Jhaveri (19), The Nephrologist by Dr. Vanessa Grubbs (20), the NephJC blog (21), and Landmark Nephrology (22). These websites were the earliest meeting places for nephrologists which invited debates and discussions. Many of these discussions moved to Twitter in ~2010. The tweets were sporadic and displaced. Hence, NephMadness was created as an attempt to focus the conversation during a discrete time point. NephMadness is an online interactive educational tool based on a single elimination tournament occuring during the month of March each year. Similar topics are pitted against each other, and a winner is chosen by a blue ribbon panel of judges. Topics are debated on social media and winners and losers and named, sparking more debate online (23). NephMadness uses the entire gamut of FOAMed; blogs, Twitter and tweetorials, Instagram, Facebook, visual abstracts, memes, podcasts, and emails list serves.

As mentioned above, Twitter has been a popular social media site for nephrology education. Twitter currently has a character limit of 280, along with the addition of hyperlinks, hashtags, pictures, and videos. Producing high-quality content in 280 characters is challenging. To overcome this, the tweetorial was developed. A tweetorial is a combination of tweets threaded (attached) together to form a series of tweets in the form of a tutorial, thus called a Tweetorial. It is defined as a “collection of threaded tweets aimed at teaching users who engage with them” (24). They help understand the pathophysiology and mechanism of the disease by answering the “why” question, which increases readers’ curiosity. The tweetorials may include polls, links to primary resources, graphics, and short one-minute videos to make them more engaging (25).

## Twitter hashtags

With a tsunami of information and tweets available on Twitter, it becomes difficult to search for a topic of interest. Hence, tweets have hashtags for categorization. For example, #NephMadness, #ISNwebinars, #NephPearls, and so on (26). The use of hashtags aids in categorization of the content which makes it easy to search. #AskRenal is a popular hashtag created by Nephrology Social Media Collective (NSMC) faculty, which can be used to ask any nephology-related query. The @AskRenal Twitter handle is a bot programed to search for any tweets using the #AskRenal hashtag (27). The @AskRenal Twitter account then retweets the original tweet containing the #AskRenal hashtag to the entire nephrology community on Twitter. Since having a large number of followers is crucial for conversation and thus is an impediment to joining Twitter, The @AskRenal bot allows for individuals who are new to Twitter and have a small number of followers to receive high quality answers to queries. An analysis of tweets using the #AskRenal hashtag demonstrated that users who tagged tweets with the #AskRenal hashtag resulted in high yield referenced answers that were quickly replied to and were not dependent on follower count (27, 28).

Nephrology Journal Club (NephJC) is a biweekly nephrology online journal club that occurs on Twitter. It is an hour-long discussion about the latest literature occurring in two time zones. A blog post serving as an editorial is published before the discussion accompanied with a visual abstract. Anyone can join the discussion by using the hashtag #NephJC. Often, the author of the publication participates in the chat. Users directly post queries related to the study and get instant answers. Regular participation in journal clubs helps to build networking relationships and collaboration (22). Many NephJC chats are then featured as a publication in the journal *Kidney Medicine* (29, 30).

Everyday cases in nephrology (ECNeph), using the hashtag #ECNeph, is a discussion of everyday cases in nephrology by nephrologists in India and across the globe. A fascinating case history slowly unwinds, keeping participants involved with active discussion about the case. It is a monthly, hour-long discussion on Twitter. Participants can participate and discuss using the hashtag #ECNeph (31).

## Twitter polls

Embedded polls allow for participants to interact with tweets. Several examples in nephrology have successfully used Twitter polls to teach. The editors of NephSIM used Twitter polls to teach acid-base disorders and examples of others using them for point of care ultrasonography in nephrology (32).

## Tweeting the meeting

Prior to 2017, conference content at ASN Kidney Week was limited to people who could physically attend, which was difficult due to financial constraints and geographical distance. Moreover, many conferences had strict policies that prohibited sharing of meeting content outside of the venue. This changed in 2017 as a result of policy change originating from the ASN Media and Communications Committee (33). This ushered a shift in which many conferences followed suit, actively encouraging the sharing of conference presentations. Thus, many conferences now use hashtags to increase engagement and enhance dissemination. Name tags are now provided with Twitter handles. A person attending one session can now follow what is happening in the other session by following the hashtag of the conference name (#KidneyWk for example). Those who are far away and cannot attend, can just follow the tweets with the help of conference hashtag and stay connected and uptodate with the latest innovations in the field. Some examples are #ISNFrontiers #ISNWCN #ERAEDTA #KidneyWk, #NKFClinicals, #KIDNEYcon (33, 34).

## Video contents

Video contents in the form of lectures, chalk talks, live and recorded webinars are accessible from YouTube and Vimeo. Glomcon has a web-based, clinical video case conference with peer group discussion involving nephrologists, nephropathologists, trainees, basic science, and clinical scientists (35). In collaboration with other societies, WIN-India, organizes live webinars, panel discussions, and debates regularly which gives an opportunity for live participation and the videos are also posted YouTube for later consumption (36, 37). WIN India strives to support women to advance their careers by providing mentorship, promoting research and collaboration (32). ISN conducts a Nuances in Nephrology live webinars on nephrology topics where the members can interact with the expert speaker and clarify the queries (38). Similar websites include NephroPOCUS.com (39) which advocates for point of care ultrasound based physical examination, “Washington University in St. Louis nephrology teaching series” (40) and “Arkana Live” (41) - videos that discuss nephropathology, “John Roberts” (42) videos on YouTube - a series for pencasts catering to medical students and internal medicine residents.

## Podcasts

It might take a lot of work for a busy nephrologist to find time to read articles, journals, books, and blog posts to stay up-to-date. With access to any personal digital device, one can access a universe of podcasts at one’s fingertips. Podcasts are a very efficient way to consume information while you are on the go - during a commute to the workplace, during a lunch break and in between meetings (43, 44). There are several podcasts for nephrologists to choose from are listed in the Table. Additionally, ISN is using Twitter space to discuss high yield nephrology topics with experts, which can be attended by any of the registered Twitter users (**Table 1**).

**Table 1:** List of Nephrology Podcasts.

Podcast Name	Launch Date	Group	Subject
ASN Podcast	2009	ASN	General Nephrology, Policy
CJASN	2017	ASN	Nephrology Advances
The Sediment	2017	American Society of Pediatric Nephrology	Pediatric Nephrology
Throwback Thursdays	2017	Arkana, Fred Silva	Nephropathology, History of Nephrology
NephTalk	2017	Satellite Healthcare	General Nephrology
Life as a Nephrologist	2018	NKF	Nephrology Careers
Freely Filtered	2019	NephJC	Nephrology Advances
Global Kidney Care	2020	ISN	General Nephrology
Kidney360	2020	ASN	Nephrology Advances
JASN	2020	ASN	Nephrology Advances
Kidney Essentials	2021	Sarah E Young, Sophia L Ambruso, Judy Blaine	General Nephrology
Channel Your Enthusiasm: The Burton Rose Book Club	2021	NephJC	Acid Base, Electrolytes, General Nephrology
Nephrology Nursing Perspectives	2021	ANNA	Nephrology Nursing
Fast Facts Nephrology	2021	Karger	General Nephrology
NANT	2021	National Association of Nephrology Technicians/Technologists	Nephrology Technicians
The Nephron Segment	2022	Samira Farouk, Matthew A. Sparks, Sam Kant, Elinor Mannon	General Nephrology
The Kidney Chronicles	2022	Emily Zangla, Annie Kouri	Pediatric Nephrology
Kidney Commute	2022	NKF	General Nephrology
Let’s Talk About Kidneys	2022	Dallas Nephrology Associates	General Nephrology
The PD Exchange	2022	Peritoneal Dialysis International	Nephrology Advances, Peritoneal Dialysis

## Visual abstracts

A visual abstract is a visual summary of key points of the journal article. Similar to the abstract section of a journal article, it summarizes the article with essential points. The purpose is to create interest in readers to read the full article. The visual abstract is not a substitute for reading the full article. In the fast-moving digital world, we have just a few seconds to capture the audience’s attention, and high-impact visual abstracts serve this purpose. A study by Ibrahim et al., in 2017 showed that visual abstracts had 7-fold higher impressions and 3-fold higher website visits compared to text-only tweets (45). Oska et al. conducted a prospective case-control cross over study where 40 research articles were tweeted in 3 formats; citation only, citation with key figure, and citation with visual abstract. The results showed that tweets with visual abstracts had twice as many article views as those that contained citations alone and citations with key figures (46).

## Emails

Email listservs are one of the first forms of two-way real time communication that developed between educators in nephrology (47). One of the first examples was the ASN Renal Educators Listserv was started in 2011 as a way for educators to communicate and share ideas (48). Even though it is an older technology, of late, it has been increasingly used to deliver newsletters directly to the recipient. One need not search the internet for content; the blog posts get directly delivered to the subscriber’s email account. The release of new episodes of podcasts, reminders of upcoming webinars, publications of new journal articles, and visual abstracts are delivered to the subscribers immediately by email (49).

## Memes

A meme is visual which is witty, ironic, gains popularity instantaneously, and spreads quickly *via* social media platforms. They were started in the middle of the 21st century and have become a global internet cultural phenomenon. A study by Vivekananth et al, compared physiology students who created memes with those who created concept maps to learn complex topics. They inferred that memes ignited interest in the subject, increased peer interactions, enhanced retention of concepts, simplified complex topics, and created a positive learning environment (50).

## Interactive learning

**Figure 2:**
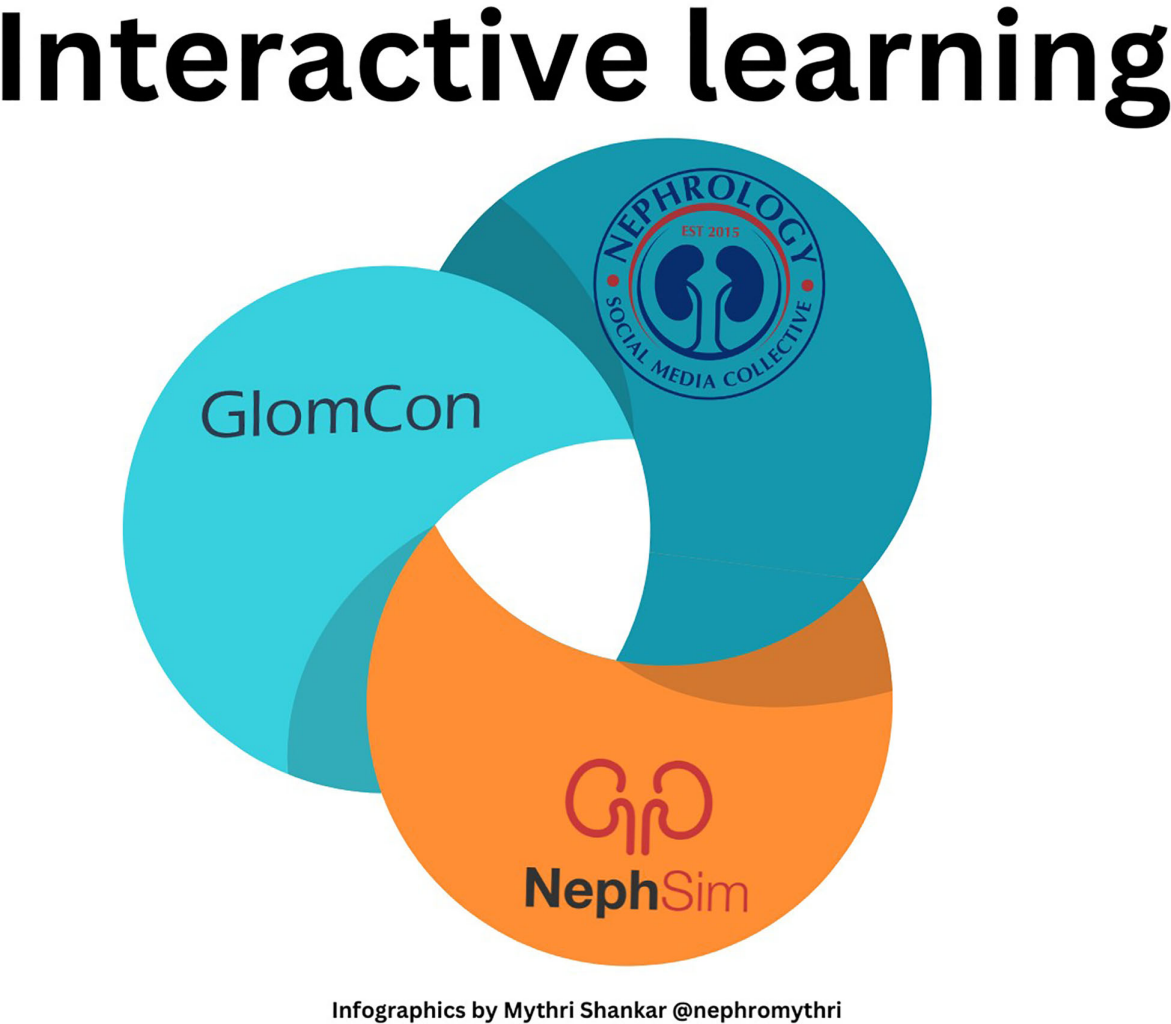
Interactive learning.

The NSMC internship (launched in 2015) is a year-long, mentored program where interns (ranging from medical students, residents, and attendings) participate in a curriculum of activities, lectures, and capstone projects to increase confidence, proficiency, and knowledge in social media. The NSMC internship is currently composed of 4 rotations; graphical communications, podcasting, NephJC Twitter journal club, and blogging/tweetorial. Modern communication skills are taught during this internship, which empowers interns to create FOAMed and become future leaders in medicine (51) (**Figure 2**).

The Glomcon fellowship (launched in 2020) admits fellows to be educated in diagnosing and managing glomerular disease from experts worldwide.The fellows present their assignments and cases, followed by an interactive discussion with expert nephrologists (52).

The International Society of Nephrology (ISN) American Nephrologists of Indian Origin (ANIO) clinical nephropathology certification program is another such year-long online program. It comprises of recorded and live videos to learn the basics of nephropathology (53).

NephSIM is a mobile-optimized tool that uses interactive nephrology cases and real time feedback to teach pathophysiology, diagnosis and management of the case (54). Additionally, NephSIM Nephrons (Launched in 2021) is a year-long mentorship program tailored for trainees who are interested in pursuing a career in nephrology (55).

## Global nephrology community

The ASN has a forum for members called ASN Communities aiming to provide a meeting place for nephrologists to discuss cases, debate controversies, and notify any upcoming opportunities for members (56). The forum also provides opportunities for educators to discuss upcoming challenges and strategies (57).

## Balancing gender inequities

Gender inequities are known to impact a woman’s career and advancement negatively. Social media platforms have helped achieve gender parity in a significant way. It provides access to mentorship, education, and research. Creating a community such as WIN, which advocates for gender equity, has played a significant role (37). The goal is to nurture interest in nephrology for young doctors and implement patient advocacy programs as well. It provides a platform for young women nephrologists to interact with senior women nephrologists and seek mentorship and collaboration (36, 37, 58). It is imperative that the social media community continue to ensure gender balance and diversity.

## Guide for first time social media users

1. Users should choose one platform that suits them the most based on the desired content and target audience (eg., Facebook, Twitter, Instagram, YouTube, LinkedIn). Similar to medicine, one should have a personalized approach, and it is best to keep professional and personal social media accounts separate as much as possible. However, one should recognize that this is challenging to accomplish completely. The level of engagement can range from passive viewing to the active creation of FOAMed content. Initially, one may find it intimidating, but as the comfort level increases, the engagement level also increases. Based on our experience, the nephrology social media community (#NephTwitter) is warm, welcoming, uplifting, and non-threatening (59).
2. Be cordial and support your peer group by sharing content created by them. Always use high-quality images and videos. Provide credit and provide appropriate citations. Join Twitter chat groups such as #NephJC, #ECNeph to learn and network (59).
3. Take breaks from social media. Strategies are to use social media during certain times of the day or certain days of the week. According to a study by Pirdehghan et al, social media use can lead to anxiety, insomnia, addiction and fear of missing out (60).
4. Follow people with shared professional interests, experts, and leaders in nephrology. It is essential to stay focused and not get carried away with distractor tweets such as politics, movies, and so on. However, it must be noted that your social media feed is your feed and it should be curated to how you want to use it.
5. Create a social media scholarship portfolio describing the content created and the associated analytics, which can be critically assessed for improvement of the content and published. The scholarship portfolio can help in job placements and promotions.

## The future

Worldwide, engagement of nephrologists with social media continues to increase (4). Many are using social media for educational purposes, and more established institutions are beginning to recognize it for promotion and tenure (61, 62). Many international nephrology organizations are investing in social media. ISN organized online coverage of the World Congress of Nephrology (WCN) in 2017. Since then, the WCN conferences have been live-streamed. The ASN has created ASN Communities to capture the interaction between its members across the globe. The University of California, San Francisco, has a rotation where a medical student reviews the medical entries to the Wikipedia website on Web 2.0 (63). The Duke University Clinical Research Training Program offers a Masters degree course titled Scientific Communication where physicians and researchers learn modern communication skills such as social media and visual abstract creation (64). Beth Israel Deaconess Medical Center offers a digital education curriculum for 2nd and 3rd year residents in the internal medicine residency program (65). The future will see more programs focused on social media education aimed at trainees.

Platforms such as TikTok, Instagram, and Facebook are still ripe for innovation, and they have a lot of potential to be explored with a robust audience. Two-way communication is limited in these social media platforms, which could be a pitfall with scope for improvement (15). Newer platforms, like Mastodon, are also gaining popularity and could threaten the popularity of Twitter.

Nephrology has been the leader in FOAMed. Intensivists, cardiologists, and endocrinologists are catching up. We should take advantage of our early start and continue to lead others in FOAMed. While uptake of social media has been swift, there is a an urgent need and important opportunity for educational research to assess the impact of social media in education (8).

## Limitations

While the use of social media for education has many positive attributes, it is important to consider the negative consequences. Using social media for education can be a double-edged sword. The posted content is often not peer reviewed; hence, there is a threat of dissemination of inaccurate and/or commercially biased information. Fact checking and feedback is provided by a robust network of experts in the audience (16). However, loud voices can sometimes drown out other perspectives. One can directly communicate with the author and corrections can be performed immediately, this bypasses the red tape which requires a structured letter to the editor. The feedback may be sporadic and imperfect but at least it’s transparent and accessible. Several scoring systems have been studied to critically appraise blog posts and podcasts, especially in critical care medicine (66). The nephrology online community has yet to adopt these metrics.

While tweeting educational material such as imaging, laboratory values, urinary sediment examination, pathology images, and case history, it is essential to obtain patients’ consent and/or ensure confidentiality. Patients may not be willing to share their data online as it may breach their confidentiality (67). Although the content is purely for educational purposes, other people, such as the patient themself, the patient’s family members, may find it offensive. Even if one deletes a tweet, someone might have already downloaded the image and reposted it later. Hence, watermarking the copyright holder on the images is one way to prevent the misuse of images. Also, one should remember that they represent their institution and remain courteous in conversations. There are instances where people have lost their jobs due to insensitive remarks made on social media platforms. An essential mantra for online users is to remember that “once an image or tweet is released, it is in the public domain forever.” (66) While it is common to place statements like, “tweets are my own and do not represent my employer” on Twitter or social media profiles, it must be noted that individuals can never completely dissociate their online behavior from their employer, family, or peer group. Thus, it is important to maintain a high degree of professionalism when engaging in social media.

Another inevitable limitation of social media is leaving a public data trail. Our online behavior is being constantly tracked and monitored by marketing companies to sell their products or political influencers to increase their vote base (68).

Despite all the limitations, nephrologists can use social media to their advantage.They can use it to reach and collaborate with the target audience. With consistent and focused creation of FOAMed, one can gain visibility and credibility. This, in turn, can open up offline opportunities, such as an invitation to become speakers, editors, research collaborations, and publications, ultimately advancing one’s career (59). Meeting nephrologists from around the world, sharing ideas, forming teams and collaborations is a huge benefit and much of our current landscape emerged from such online relationships (for example NephJC led to NephJC Freely Filtered and so on). Not to forget, people’s clinical and research careers have started through just online messages.

## Conclusion

Medical media is ever-evolving. There is a growing interest in nephrologists to consume educational content from social media and create and disseminate original content. There is increasing involvement of nephrologists in real-time debates, discussions, and analysis of data presented. With the growing interest, knowing how to use social media platforms effectively to one’s leverage is crucial. Lastly, it is time to study how social media and FOAMed have impacted education in both the short and long term. The nephrology community should continue to invest money, time, and energy into research, best practices, and the creation of new content.

## Author contributions

MS conceptualized and wrote the article. MAS edited and reviewed the document. Both authors contributed to the article and approved the submitted version.
